# The effect of mimicking febrile temperature and drug stress on malarial development

**DOI:** 10.1186/1476-0711-8-19

**Published:** 2009-06-12

**Authors:** Ratchaneewan Aunpad, Sangdao Somsri, Kesara Na-Bangchang, Rachanee Udomsangpetch, Mathirut Mungthin, Poom Adisakwattana, Wanna Chaijaroenkul

**Affiliations:** 1 Graduate Program in Biomedical Sciences, Faculty of Allied Health Sciences, Thammasat University, Pathumthani, Thailand; 2 Department of Pathobiology, Faculty of Science, Mahidol University, Bangkok, Thailand; 3 Department of Parasitology, Phramongkutklao College of Medicine, Bangkok, Thailand

## Abstract

**Background:**

Malaria remains one of the most important tropical diseases of human with 1–2 million deaths annually especially caused by *P. falciparum*. During malarial life cycle, they exposed to many environmentally stresses including wide temperature fluctuation and pharmacological active molecules. These trigger malarial evolutionarily adaptive responses. The effect of febrile temperature on malarial growth, development and drug susceptibility by mimicking patient in treatment failure before and after drug uptake was examined.

**Methods:**

Sensitivities of *P. falciparum *to antimalarial drug (chloroquine, mefloquine, quinine and artesunate) were investigated based on the incorporation of [^3^H] hypoxanthine into parasite nucleic acids or radioisotopic technique. The number of parasites was examined under microscope following Giemsa staining and the parasite development at the end of each phase was counted and comparison of parasite number was made. The proteome was separated, blotted and hybridized with anti-Hsp70s primary antibody. The hybridized proteins were separately digested with trypsin and identified by MALDI-TOF peptide mass fingerprint.

**Results:**

The results show that febrile temperature is capable of markedly inhibiting the growth of field isolate *P. falciparum *but not to K1 and 3D7 standard strains. K1 and 3D7 grown under heat shock developed greater and the reinfection rate was increased up to 2-folds when compared to that of non-heat shock group. The IC_50 _value of K1 toward chloroquine, mefloquine and quinine under heat shock was higher than that of K1 under non-heat shock which is opposite to that of 3D7. Heat shock caused death in field isolated parasite. It was also found that the febrile temperature coped with chloroquine uptake had no effect to the development, drug sensitivity and the parasite number of K1 strain. In the opposite way, heat shock and chloroquine shows extremely effect toward 3D7 and field isolate PF91 as shown by higher number of dead parasites compared to that of control group. After culture under high temperature with artesunate, the total parasite number of all strains including K1, 3D7 and PF91 was extremely decreased and the parasite was not found at the end. Additionally, the expression of *pf*Hsp70s was found in all strains and conditions as shown in 120 kDa hybridized band. However, the proteome extracted from K1 grown under heat shock with chloroquine, anti-*pf*Hsp70 interacted with additional three bands identified by MALDI-TOF as elongation factor-1α (83 kDa), pf*Hsp*86 (60 kDa) and phosphoethanolamine *N*-methyltransferase (43 kDa).

**Conclusion:**

In conclusion, febrile temperature was capable of markedly inhibiting the growth of field isolate *P. falciparum *while the development, reinfection rate and drug (chloroquine, mefloquine and quinine) resistant level of standard strain K1 was enhanced. However, the febrile temperature coped with chloroquine had no effect to the development, drug sensitivity and the parasite number of K1 strain. In the opposite way, heat shock and chloroquine showed extremely effect toward 3D7 and field isolate PF91 as shown by some died parasites. Heat shock protein 70 (*pf*HSP70) of strain K1 under heat shock with chloroquine might involved in many pathways in order to sustain the parasite.

## Background

Malaria remains one of the most important diseases of man with over half the world's population at risk of infection and 1–2 million deaths annually. There are four different species of human malaria parasites namely, *Plasmodium falciparum*, *P. vivax*, *P. malaria*, and *P. ovale*. *P. falciparum *is the most dangerous malaria species as it often leads to the death and can be fatal within few hours of the first symptom [1]. The human malarial parasite *P. falciparum *interacted with wide temperature variation during its life cycle, ranging from 25°C or 26°C in the mosquito vector and 37°C in humans, to 40–41°C during febrile episodes in the patient [2]. It was found that the parasite number was decrease and parasite growth was inhibited by febrile temperature when cultured parasite in varying temperature ranging from 37 to 40°C [3]. The febrile temperatures could also synchronize parasite and inhibit the parasite growth, although the ring stage resisted to the high temperature [4]. However, the influence of such repeated exposures to high temperatures on parasite growth, development and drug susceptibility have not been studied systemically.

Heat shock protein (HSP) is mediator for cytoprotection and growth promotion [5] not only in the normal condition but also in the continuation of life under stress. The genes encoding three Hsp70 and one Hsp90 of the *P. falciparum *have been cloned [6]. The biological roles of these proteins in malaria are not fully understood but it is possible that they help the parasite through various stresses both in and out of the host [6]. The overexpression of PfHsp70 encoding gene producing higher amounts of PfHsp70 proteins are probably protect the parasite and survive *in vivo *during malaria fever [7,8]. Together with drug, the effect of febrile temperature toward parasite not only to the development, drug sensitivity but also to the heat shock protein expression is very interesting to study. These data might succor us to understand the complexity of this kind of life and overwhelm this powerful parasite.

## Methods

### Culture system for parasite maintenance

*Plasmodium falcifarum *strains 3D7 (CQ-sensitive clone), K1 (CQ-resistance clone) and 1 field isolate (PF91) collected from endemic areas of Thailand were used. They were kindly provided by Department Parasitology, Pramungkutkalo Medical College, Thailand and Malaria Research Unit, Department Pathobiology, Mahidol University, Thailand. All isolates were continuously cultured using the standard methods [9,10] with modifications. All of the test standard drug solutions were filtered through a 0.2 μm acrylic filter sterile (Gelman Sciences Inc, UK) prior to use. Complete culture medium was prepared by adding 20 ml of pooled human serum, to each 200 ml aliquot of stock RPMI1640 medium containing L-glutamine without sodium bicarbonate (Gibco URL, UK).

### *In vitro *drug sensitivity assay

Sensitivities of *P. falciparum *standard strain and one field isolate to chloroquine (CQ), artesunate (ARS), mefloquine (MQ) and quinine (QN) were investigated based on the incorporation of [^3^H]hypoxanthine into parasite nucleic acids or radioisotopic technique [11]. The level of radioactivity uptake was used as index of parasite growth.

### Effect of febrile temperature on malarial drug sensitivity

In order to investigate the effect of temperature on drug sensitivity of malarial parasites, the 5% parasite with highly synchronous ring stage *P. falciparum *(synchronized 2–4 h post-invasion) was grown under the temperature of 37°C (non-heat shock, non-HS) and 40°C (heat shock, HS). At the end, total parasite culture was collected and used for *in vitro *drug sensitivity assay.

### Effect of febrile temperature on malarial growth and development

**Figure 1:**
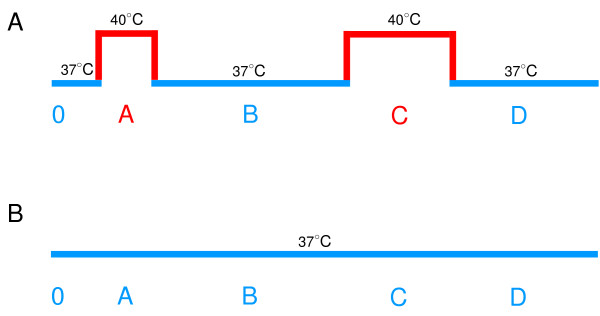
**The schematic pattern of temperature for heat shock (A) and non-heat shock group (B)**. The blue line represented the culture temperature at 37°C and the red line represented the culture temperature at 40°C.



The culture of 5% highly synchronous ring stage parasite, *P. falciparum*, (synchronized 2–4 h post-invasion) was subjected to a temperature shift pattern at 40°C (phase A) for 2 h followed by incubation at 37°C (phase B) for an additional 18 h. Then the culture was shifted to 40°C (phase C) for 4 h before turning down to 37°C (phase D) for 24 h (figure 1). The experiments performed at 37°C of all phases were used as non-HS control. The number of parasites was examined under microscope following Giemsa staining. The parasite development at the end of each phase A, B, C and D was counted and comparison of parasite number was made in order to examine the effect of temperature on overall asexual development.

### Effect of febrile temperature and drug stress on malarial growth and development

The culture of highly synchronous ring stage parasites (synchronized 2–4 h post-invasion) was grown under temperature shift patterns (figure 1) coped with each antimalarial drug, *i.e*., chloroquine and artesunate (at concentrations of 40 and 1 nM, respectively). The experiments performed at 37°C of all phases were used as non-HS control. The number of parasite, parasite development by the end of each phase was examined. The total parasite culture was collected and used for *in vitro *drug sensitivity assay as described above.

### Effect of febrile temperature and drug stress on *P. falciparum *heat shock protein 70

The parasite cells were collected at the end of phase C and washed three times in PBS buffer. Then 0.15% saponin (Sigma Aldrich, USA) with 50 μl of protease inhibitor (PMSF) was added and further incubated at 37°C for 10 min. After that, the pellet was washed three times with cool PBS buffer and sonicated at 25 Amp, on/off 3 sec for 5 times after the addition of cool 50 mM Tris-HCl pH 7.4 (Merck, UK) and 30 μl of protease inhibitor. The supernatant containing total proteins was then collected after centrifugation at 4°C (12,000 × g) for 30 min and 50 μl of protease inhibitor was added. The proteins were separated by 12% SDS-PAGE and transferred to a western blot membrane (Hybond-Cextra, Amersham, USA). The membrane was incubated with anti-Hsp70s primary antibody for 1 h and incubated with secondary antibody for 30 min. The membrane was incubated with the detection reagent (4-chloro-1-napthol, methanol and H_2_O_2_) until the colour was developed. The proteins on SDS-PAGE gel with the same location with the hybridized bands were separately digested with trypsin and identified by MALDI-TOF peptide mass fingerprint. The data was searched through PMF database search. The proteins identifications were done by Bioservice Unit (BSU), NSTDA Building, Thailand.

### Data Analysis

Distribution of the data (number of parasite counts) obtained from all experiments during all phases will be assessed by Komogolov Sminov test. Comparison of the difference in number of parasite counts between two groups (HS and non-HS) will be performed using paired t-test or Wilcoxon Signed Rank test, where appropriate. Statistical significance level will be set at α = 0.05 for all tests.

## Results

### *In vitro *drug sensitivity assay

The level of chloroquine (CQ), quinine (QN), mefloquine (MQ) and artesunate (ARS) susceptibility was determined as resistant or sensitive by using the criteria of Pickard and colleague [12]. The IC_50 _value of standard strain K1 used in this study against CQ, ARS, MQ and QN was 100.511, 314.982, 10.940 and 0.424 nM, respectively whereas the IC_50 _value of strain 3D7 against CQ, ARS, MQ and QN was 11.332, 47.117, 18.208 and 0.434 nM, respectively. The field isolate PF91 showed the IC_50 _against CQ, ARS, MQ and QN at 63.585, 37.886, 8.124 and 1.254 nM, respectively. There was no evidence indicating ARS resistant therefore ARS susceptibility was not classified.

### Effect of febrile temperature on malarial drug sensitivity

The strain K1, 3D7 and PF91 were tested for their susceptibilities to CQ, ARS, MQ and QN when grown under temperature stress or heat shock (HS) at 40°C for 2 h. The results showed that the IC_50 _value of K1 strain grown under HS and non-heat shock (non-HS) was not different whereas the IC_50 _value of strain 3D7 grown under HS was lower than that of non-HS (Table 1). The effect of temperature on drug sensitivity (IC_50_) after heat (2 h) followed by incubation at 37°C for an additional 18 h (new ring infection, NRF) showed the same result to that of without NRF.

**Table 1:** The drug susceptibility (IC_50 _value) of standard strains (K1 and 3D7) and field isolate PF91 after heat shock (HS) and non-heat shock (non-HS) condition.

**Parasite strain**	**Mean IC_50 _of drug susceptibility (nM)**			
	
	**CQ**	**QN**	**MQ**	**ARS**
K1 non-HS	100.511	314.982	10.940	0.424
K1 HS	104.468	300.071	11.259	0.862

3D7 non-HS	11.332	47.117	18.208	0.434
3D7 HS	4.722	43.936	15.470	0.660

PF91 non-HS	63.585	37.886	8.124	1.254
PF91 HS	CC	CC	CC	CC

K1 non-HS (NRF)	100.511	314.982	10.940	0.425
K1 HS (NRF)	104.468	300.072	11.260	0.862

3D7 non-HS (NRF)	11.332	47.118	18.208	0.435
3D7 HS (NRF)	4.722	43.937	15.470	0.661

PF91 non-HS (NRF)	63.586	37.886	8.125	1.254
PF91 HS (NRF)	63.586	37.886	8.125	0.954

NRF represents new ring infection when the parasite was cultured back to 37°C for one cycle.

### Effect of febrile temperature on malarial growth and development

**Figure 2:**
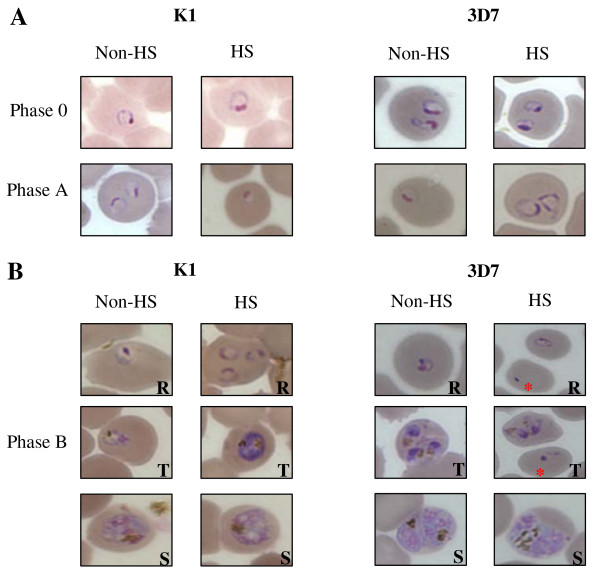
**The Morphology of K1 and 3D7 strain under non-heat shock (non-HS) and heat shock (HS) at phase 0, phase A and phase B**. Figure 2A shows the parasite at ring stage of phase 0 and phase A. Figure 2B shows the parasite development in three different stages; ring (R), trophozoite (T), schizonts (S). The red star indicates died parasite.

The laboratory strain K1 and 3D7 used in this study contained only young intraerythrocytic parasites (ring) as shown in figure 2A. The results showed that at phase 0, the parasites were at the same stage even in the HS group after incubation at 40°C for 2 h. The parasite number of K1 and 3D7 strains was not decreased. At phase 0, the number of K1 and 3D7 parasite in both HS and non-HS group after incubation at 40°C strains was 5 × 10^5 ^parasite infected red blood cell (PIRBC)/1 μl pack red blood cell (PRBC) (4ure 2).

**Figure 3:**
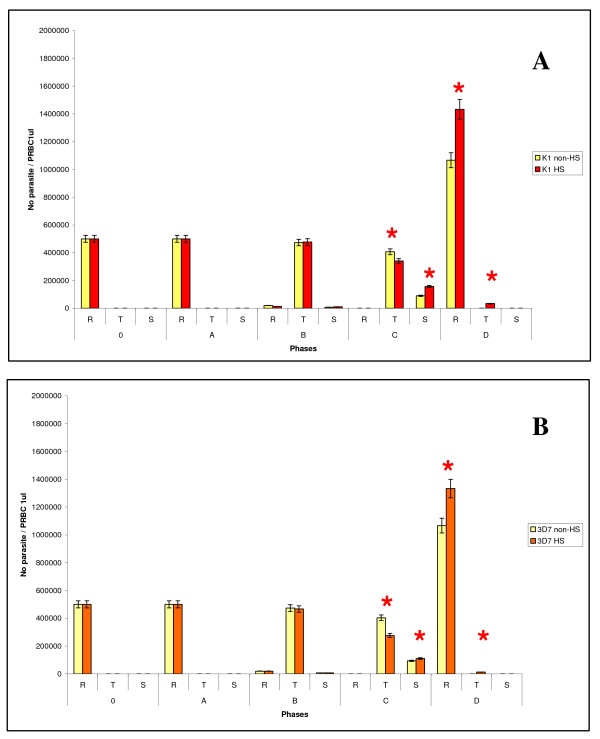
**The graph shows the number of parasite in different stages of each phase**. Figure A and B show the parasite number of K1 and 3D7 strain, respectively. The red star indicates that the parasite numbers were significantly different at 95%CI.

When the temperature was down from 40°C to 37°C for 18 h (phase B), K1 strain grown under HS and non-HS could maintain the total number of parasites at 5 × 10^5 ^PIRBC/1 μl PRBC (*p *= 0.423 at 95% CI). The total number of parasites of 3D7 under HS was decreased from 5 × 10^5 ^PIRBC/1 μl PRBC (non-HS) to 4.9 × 10^5 ^PIRBC/1 μl PRBC (HS). In addition, the morphology of K1 under HS and non-HS at phase B was the same whereas some parasites of strain 3D7 under HS died (figure 2 and 3).

**Figure 4:**
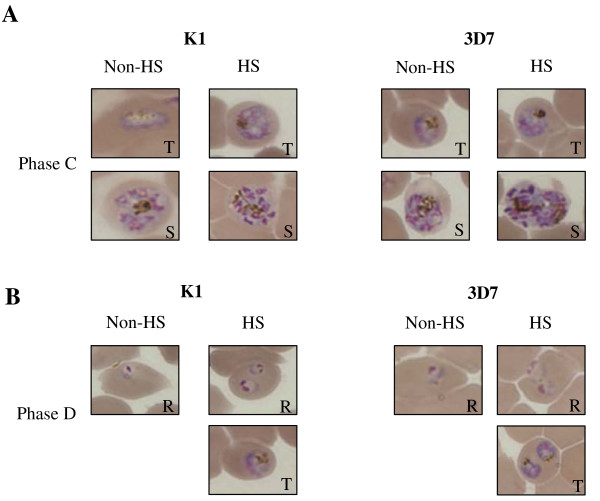
**The morphology of K1 and 3D7 strain under non-heat shock (non-HS) and heat shock (HS) at phase C (A) and phase D (B)**. The parasites at trophozoite and schizonts stage are shown in phase C. The parasite reinfection and development in new cycle; ring (R) and trophozoite (T) are shown in phase D.

At phase C, the HS group was exposed to 40°C for 4 h after medium changing at the end of phase B. During phase C, the parasites were at trophozoite stage. At the end of phase C, the total number of K1 grown under HS and non-HS was the same whereas the total number of 3D7 grown under non-HS and HS were significantly different (*p *= 0.001 at 95% CI). The morphology of K1 and 3D7 parasite of non-HS and HS was not different (figure 4).

At the end of phase C, the parasite was cultured at 37°C for 24 h (phase D) and the reinfection rate and development were determined (Table 2). The parasite grown under HS of both strains developed to trophozoite while the parasite grown under non-HS group did not develop to tropozoite (figure 3).

**Table 2:** The reinfection ratio of K1 and 3D7 strain of both non-HS and HS group.

	Reinfection ratio	*p *value at 95% CI between non-HS and HS
K1 non-HS	2.1456	0.04
K1 HS	2.9863	

3D7 non-HS	2.1483	0.05
3D7 HS	3.4870	

The *p *value represents the correlation between non-HS and HS which is significantly different.The *p *value represents the correlation between non-HS and HS which is significantly different.

### Effect of febrile temperature and drug stress on malarial growth and development

**Figure 5:**
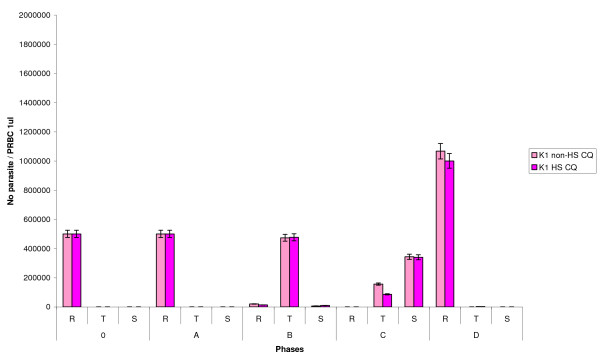
**The graph shows the number of parasite in different stages of each phase**. The parasite number of K1 non-heat shock with chloroquine (K1 non-HS CQ) and K1 heat shock with chloroquine (K1 HS CQ), respectively. R, T and S represent ring, trophozoite and schizont, respectively.

**Figure 6:**
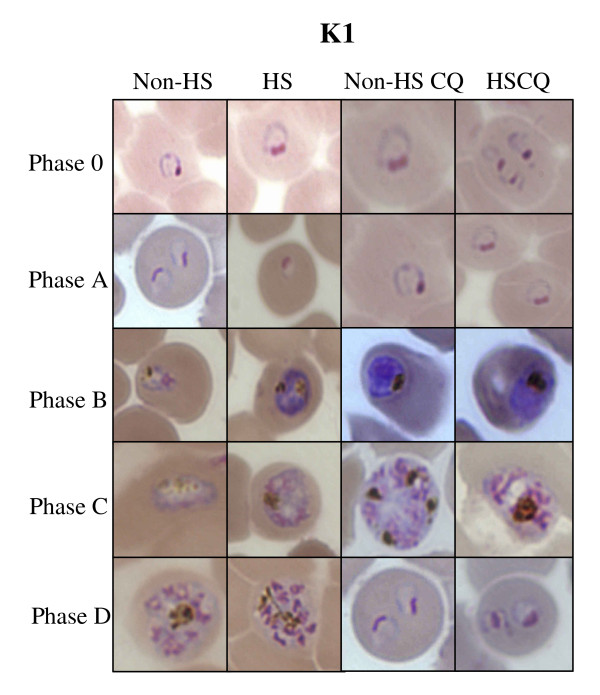
**The morphology of K1 under different conditions which are K1 under non-heat shock (K1 non-HS), K1 under heat shock (K1 HS), K1 under non-heat shock with chloroquine (K1 non-HS CQ), K1 under heat shock with chloroquine (K1 HS CQ)**. These figures show the morphology of the parasite at ring stage during phase 0 and phase A, trophozoite stage during phase B and schizont stage during phase C and D.

This experiment mimics the condition where malarial parasite grown in patient's body with treatment failure combined with fever. The strain K1 was co-cultured with 40 nM CQ and grown under non-HS and HS. The results showed that the total number of parasite was not different at phase 0, A and B while at phase C, the number of trophozoite under non-HS with CQ was higher than that of K1 under HS with CQ. During phase D, the number of schizonts was similar in all conditions whereas the number of ring stage under non-HS with CQ was higher than that of under HS with CQ. Only K1 strain under HS developed further to trophozoite (figure 5). The morphology of parasite in all conditions was almost the same. Some of K1 schizonts under HS with CQ at phase C showed some hyposegmented (figure 6). The number of trophozoite stage at phase C of K1 under non-HS and HS without CQ was higher than that of K1 under non-HS and HS with CQ. Whereas the number of schizonts stage in K1 under non-HS and HS with CQ was higher than that of K1 under non-HS and HS without CQ.

**Figure 7:**
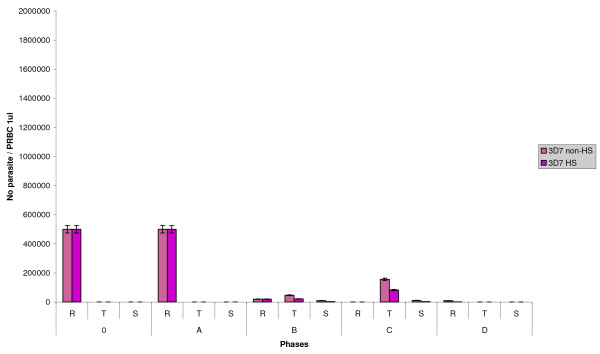
**The graph shows the number of parasite in different stages of each phase**. The parasite number of 3D7 under non-heat shock with chloroquine (3D7 non-HS CQ) and 3D7 under heat shock with chloroquine (3D7 HS CQ) were shown. R, T and S represent ring, trophozoite and schizont, respectively.

**Figure 8:**
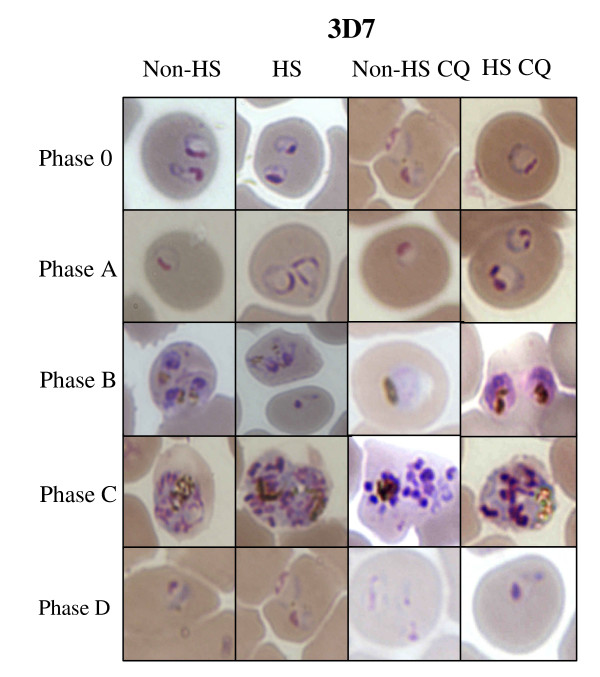
**The morphology of 3D7 parasite under different conditions which are 3D7 under non-heat shock (3D7 non-HS), 3D7 under heat shock (3D7 HS), 3D7 under non-heat shock with chloroquine (3D7 non-HS CQ) and 3D7 under heat shock with chloroquine (3D7 HS CQ)**. These figures show the morphology of the parasite at ring stage during phase 0 and phase A, trophozoite stage during phase B and schizont stage during phase C and D.

The strain 3D7 was co-cultured with 40 nM CQ and grown non-HS and HS. The results showed that at phase 0 and A, the total number of parasite was not different whereas at phase B, the total number of parasite was extremely decreased. At phase C, the total number of trophozoite under non-HS with CQ was higher than that of 3D7 under HS with CQ however the total number of schizont was the same. At phase D, the number of ring stage in 3D7 under non-HS with CQ and 3D7 under HS with CQ was very small (figure 7). The morphology of 3D7 strain in all conditions at phase 0 and A was almost the same. There were some hyposegmented schizonts found only 3D7 grown under non-HS and HS with CQ (figure 8). The number of trophozoite of 3D7 under non-HS and HS without CQ at phase C was higher than that of 3D7 under non-HS and HS with CQ. The number of schizonts in 3D7 under non-HS and HS with CQ was higher than that of 3D7 under non-HS and HS without CQ.

### Effect of febrile temperature and drug on *P. falciparum *heat shock protein 70

**Figure 9:**
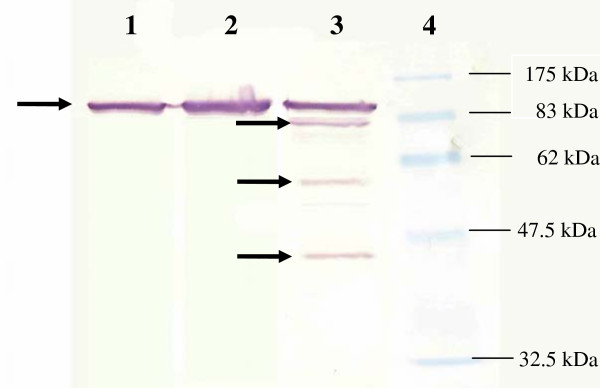
**The immunoblotting membrane of *P. falciparum *isolate PF91 (lane 1), 3D7 (lane 2) and strain K1 (land 3) grown under heat shock with chloroquine (HS CQ) conditions**. Arrows indicate four bands hybridized with *pf*HSP70 antibody with the molecular mass of 120 kDa, 83 kDa, 60 kDa and 40 kDa. Lane 4 is molecular weight protein marker.

The results showed that *pf*HSP70 antibody could bind only to the protein with the molecular mass of 120 kDa in all conditions except in K1 grown under HS with CQ condition. There were four hybridized bands with the molecular mass of 120 kDa, 83 kDa, 60 kDa and 40 kDa, respectively from the total protein of strain K1 grown under HS with CQ (figure 9).

As identified by MALDI-TOF peptide mass fingerprint (Bioservice Service Unit, NSTDA) and searched through the database, the band with molecular mass of 120 kDa in all gels was identified as *Plasmodium *heat shock protein (score 110) and *pf*HSP 70 (score 87). Three bands hybridized with *pf*HSP70 antibody (figure 9) with the molecular mass of 83 kDa, 60 kDa and 40 kDa were identified as elongation factor 1-α of *P. falciparum *(score 220), *P. falciparum *heat shock protein 86 (score118) and phosphoethanolamine *N*-methyltransferase (score 110), respectively.

## Discussion

Malaria is still one of the most important tropical diseases in human. In order to understand its complexity and combat against this kind of life, the parasite development, drug sensitivity and protein alteration of parasite under stress such as drug or febrile temperature is challenging. As indicated by *in vitro *drug sensitivity assay, the standard strain K1 used in this study was found to be chloroquine resistant but sensitive to quinine, mefloquine and artesunate whereas the strain 3D7 was classified as sensitive to chloroquine, quinine, mefloquine and artesunate. It is not surprising to find that one field isolate PF91 presented a moderate resistant to chloroquine because there was evidence showing that the parasite resists to chloroquine in Thailand [13]. There was no evidence indicating ARS resistant. In order to study the effect of stresses toward parasite, the culture of 5% parasite with highly synchronous ring stage *P. falciparum *was exposed to drug at IC_50 _concentrations but all of the parasites died. Therefore, chloroquine and artesunate concentration used in all experiments was 40 and 1 nM, respectively.

The human malarial parasite *P. falciparum *interacted with wide temperature variation during its life cycle, ranging from 25°C or 26°C in the mosquito vector and 37°C in humans, to 41°C during febrile episodes in the patient [2]. This study has examined the influence of two repeated febrile episodes on parasites growth by exposing them to elevated temperature twice, at ring and trophozoite stage (phase A and C) with two recovery phases (phase B and D). It was found that high temperature did not inhibit parasite growth in all stages of standard K1 strain and high temperature has no effect to any stage of K1 in this study. In 1989, Kwiatkowski found that ring stage parasite can resist to high temperature up to 41°C while this temperature can effect to trophozoite stage and developing schizonts were particularly vulnerable [4]. This was represented similarly to the work of Long and his colleagues in 2001 showing that the development of the parasites stopped at late trophozoite and schizonts stage within the first growth cycle at temperature above 40°C [3]. The schizonts appeared pyknotic and hyposegmented and the parasites failed to develop into new cycle. Moreover they found that the effects of hyperthermic conditions parasites growth on wild isolates were similar to those of laboratory-adaptive strain [3]. These are not in agreement with our data and it may be due to the difference in the length of time when the parasites were exposed to high temperature. The parasite cultures were exposed to a long period of high temperature up to 24 h in their experiments while the parasites was exposed twice to the short time of elevated temperature mimicking febrile episode (phase A at 40°C for 2 h and phase C at 40°C for 4 h) in our experiment. The adherence of red blood cells infected with ring stage was also increased considerably by brief heating and fever-induced cytoadherence was associated with enhanced expression of PfEMP-1 on the ring infected red blood cell surface [14]. Moreover, Pavithra and colleagues found that a prior heat shock had a stimulatory effect on parasite development during the subsequent exposure to heat shock. These observations imply that appearance of repeated febrile episodes in malaria patients can hasten intra-erythrocytic development of the parasite [5]. These are in agreement with our results. When the parasites were exposed to Phase A at 40°C for 2 h following Pavithra and colleagues in order to promote prior heat shock effect [5], the laboratory-adaptive strains, K1 and 3D7, under HS developed efficiently to trophozoite and schizonts stage at phase C (*p*K1 = 0.032, *p*3D7 = 0.042 at 95% CI) and the reinfection rate of K1 under HS and 3D7 under HS (*p*K1 = 0.04, *p*3D7 = 0.05 at 95% CI) was also promoted. The previous research found that the effect of hyperthermic conditions on wild isolated parasites growth were similar to those of laboratory-adaptive strain [3]. Our data shows that the survival rate of field isolate PF91 was reduced (*p *= 0.05 at 95% CI). The effect of temperature on morphology of field isolated parasites was found similarly to that of Oakley and his colleagues [15]. The temperature promoted the presence of pyknotic form or "crisis form" which is the appearance of parasites undergoing death [15]. The appearance of crisis form was significantly evident following 4 h of culture at 41°C [15] whereas the crisis from of field isolate PF91 was found after heating for 2 h following by incubation at 37°C. These results are not found in K1 standard strain but trifling appeared in 3D7.

The effect of temperature on drug susceptibility of parasites was tested to the parasites exposed to heat at ring stage for 2 h followed by drug sensitivity tests immediately. The result indicated that IC_50 _value to chloroquine, mefloquine and artesunate of strain K1 under HS was higher than that of K1 under non-HS significantly (p = 0.04 at 95% CI). The IC_50 _value of K1 under non-HS with quinine was higher than of K1 under HS. The effect of temperature on K1 drug susceptibility was similar to that of 3D7 only toward artesunate. It was found that IC_50 _value to artesunate of 3D7 under heat shock was higher than that of 3D7 under non- HS condition (p = 0.04 at 95% CI). The IC_50 _value to chloroquine, quinine and mefloquine) of 3D7 under non-HS was higher than that of 3D7 under HS (p = 0.04 at 95% CI). The IC_50 _value of field isolate under HS could not be calculated because heat shock causing death in parasites.

The IC_50 _value of K1 under HS was higher than that of K1 under non-HS whereas the IC_50 _of 3D7 under non-HS was higher than that of 3D7 under HS This might be explained by the effect of high temperature toward drug transporter reported by Cecilia and her colleagues in 1997 [16]. The chloroquine uptake by *P. falciparum *infected erythrocyte is temperature-dependent and saturable and the existence of a specific parasite-encoded protein that facilitates chloroquine uptake. For strain with chloroquine resistant, the temperature does not effect to chloroquine uptake whereas in sensitive strain, temperature effects to chloroquine uptake. The increasing in temperature increases chloroquine uptake [16]. Although, there was no report in the effect of high temperature toward mefloquine, quinine and artesunate, high temperature might have some effects to drug transport or drug mechanism by direct or indirect way as shown in our study. When the parasites were heated at 40°C for 2 h and cultured back at 37°C until new ring infected (NRF), the IC_50 _value of strain K1 and 3D7 was similar to that of heating at ring stage for 2 h followed by drug susceptibility testing immediately. The IC_50 _of field isolate PF91 under HS and non-HS was not significantly different (*p *= 0.5 at 95% CI). Therefore, high temperature shows some effects only toward the drug susceptibility of standard or laboratory strain K1 or 3D7 but not to field isolate.

In order to observe the effect of temperature and drug by mimicking the situation of treatment failure in malaria patients coped with fever, it was found that high temperature had no effect to the development of parasite with chloroquine resistant (K1 strain). When K1 strain was cultured with 40 nM drug under heat at ring stage for 2 h (phase A), parasite developed to phase B similarly to the other groups grown under HS or non-HS with or without drug when compared by the total number of parasite between these groups. These might be due to the concentration of chloroquine used which is lower than IC_50 _of K1 strain therefore the drug can not harm the parasite. Additionally, K1 is chloroquine resistant strain and it might have some proposed mechanisms such as increasing in vacuolar pH, enhancing in drug efflux, reducing in drug binding, losing in chloroquine transporter and changing in glutathione-S-dehydrogenese (GSH) protein. After culture parasites at phase B (37°C) for 18 h followed by heating again at 40°C for 4 h, the resistant strain was tolerant to high temperature by comparing the total parasite number between these groups. However, almost parasites developed to schizont and trophozoite. After culture parasites at phase D, the data indicated that temperature did not effect to the reinfection rate of the parasite as compared by the number of parasite at phase D.

The total parasite number of strain 3D7 between non-HS and non-HS with chloroquine after phase B was significantly different (*p *= 0.05 at 95%CI). This data illustrates the effect of drug toward 3D7 development. The total number of parasite between 3D7 under HS and under HS with chloroquine was also significantly different (*p *= 0.05 at 95%CI). This might be due to the increase in chloroquine uptake [16]. Moreover, the total number of parasite between non-HS with chloroquine and HS with chloroquine was significantly different (*p *= 0.05 at 95%CI) indicating that temperature might also increase chloroquine drug uptake of the parasite [16]. The total number of parasite at phase C and D between these groups are significantly different. Therefore, high temperature increases drug uptake and also coped with second heat shock might induce higher drug uptake in the parasite [16].

The field isolate is moderate resistant to chloroquine and also the temperature has extremely effect toward parasite development. This might explain the reason why almost parasite after phase A can not develop to phase B. Moreover, this might be due to the drug concentration which is close to IC_50 _value coping with another factor that is high temperature. These might promote more drug uptake into the parasite and more parasites died showing the direct effect of temperature.

When culture under high temperature with artesunate, the total parasite number of all strains including K1, 3D7 and PF91 was extremely decreased at phase B and the parasite was not found at phase C and D. The decrease in the number of parasite in phase B, K1 (*p *= 0.01 at 95% CI), 3D7 (*p *= 0.005 at 95% CI), field isolate PF91 (*p *= 0.01 at 95% CI) might be from the effect of artesunate coped with temperature. This might indicate that all parasites died at phase C or the sensitivity of this method is not enough to detect. There was no parasite reinfection during phase D. This might be from two reasons, first is the level of the parasite after phase C was too low to detect by the conventional method and the second is parasite died since phase B.

It was found that anti-*pf*HSP70 binds to the proteins extracted from all conditions. Not surprisingly, two chaperones, HSP70 and 90 orthologs, which have been implicated in the heat shock response across the phylogenetic spectrum of life [15] meaning that *pf*Hsp70s are stress response that will be expressed when the parasite exposed to the stress such as high temperature or drug. The proteome extracted from K1 grown under heat shock with chloroquine, anti-*pf*Hsp70 interacted with four bands whereas anti-*pf*Hsp70 interacted with only one band in another strain under different conditions. After protein analysis by MALDI-TOF peptide mass fingerprint, the 120 kDa band from all conditions is identified as heat shock protein 70 or pfHSP70s. Higher molecular weight might be caused by the complex formation of heat shock protein as molecular chaperone. The *pf*Hsp70 are chaperone complex consisting of *pf*Hsp 90, PfPP5, tubulin and an additional protein that is unidentified. The complex was similar to that of higher eukaryotes both in term of size as well as composition [5]. Normally, *pf*Hsp 70 have six homolog which are *pf*Hsp70-1 with molecular mass of 74 kDa [6],*pf*Hsp70-2 with molecular mass of 78 kDa [7], *pf*Hsp70-x with molecular mass of 76 kDa [17], *pf*Hsp70-3 with molecular mass of 73 kDa [6], *pf*Hsp70-z with molecular mass of 100 kDa and *pf*Hsp70-y with molecular mass of 108 kDa [17]. The 83 kDa hybridized band was identified as elongation factor 1-α (EF-1α). From the previous research studied in rat brain, it was found that heat shock factor 1 (HSF-1) correlated with Hsp70, Hsp 27 and Hsp 90 was found after heating at 41°C. HSF-1 probably binds to Hsp70 and then EF-1α binds to this complex to undergo phosphorylation [18]. The parasite might release EF-1α with direct availability for complex formation with HSF-1 and heat shock receptor (HSR) in the nuclease. Temperature and drug stress possibly changes the capacity of EF-1α to form a complex with other components of the initiation complex. These are major possibilities that will be explored in the future. The 60 kDa hybridized band was identified as *pf*Hsp 86. *pf*Hsp 86 is the one of HSP 90 homolog which is normally binding with Hsp70 to form a functional complex [19]. The 40 kDa hybridized band was identified as phosphoethanolamine *N*-methyltransferase. This enzyme catalyzes the transfer of a methyl group from S-adenosyl-L-methionine to the amino group of the tetrahydrobenzylisoquinoline alkaloid coclaurine [20,21]. This is a unique *N*-methyltransferase in the biosynthesis of benzylisoquinoline alkaloids. The previous studies did not present any correlation between heat shock protein and this enzyme so far. The reason why *pf*Hsp70 could bind to this protein will be explored in the future.

## Conclusion

Malaria remains one of the most important tropical diseases of man with 1–2 million deaths annually which mainly caused by *P. falciparum *[1]. In order to understand the complexity of this kind of life, the parasite development, drug sensitivity and protein alteration of parasite under stresses such as drug or febrile temperature is challenging. This will lead to the way to combat this powerful parasite. The standard strain K1 used in this study was found to be chloroquine resistant but sensitive to quinine, mefloquine and artesunate whereas the strain 3D7 was classified as chloroquine, quinine, mefloquine and artesunate sensitive. The field isolate PF91 presented a moderate resistant to chloroquine. The drug chloroquine and artesunate concentration used in all experiments was 40 and 1 nM, respectively.

The human malarial parasite *P. falciparum *interacted with drug stress and also with wide temperature variation during its life cycle. The standard strains, K1 and 3D7, under heat shock (HS) developed efficiently to trophozoite and schizonts stage at phase C and the reinfection rate of K1 under HS and 3D7 under HS was also promoted. The appearance of crisis form of field isolate PF91 was found after heating for 2 h following by incubation at 37°C. These were not found in K1 standard strain but trifling appeared in 3D7 strain.

High temperature had effect to malarial drug sensitivity in K1 strain different from that of 3D7 strain. The IC_50 _value of K1 toward chloroquine, quinine and mefloquine under HS was higher than that of K1 under non-heat shock (non-HS) whereas the IC_50 _of 3D7 toward to chloroquine, quinine and mefloquine under non-HS was higher than that of 3D7 under HS. The effect of temperature on K1 drug susceptibility was similar to that of 3D7 only toward artesunate. The febrile temperature caused death in field isolate therefore the IC_50 _value of under HS could not be calculated. This might be explained by the effect of high temperature toward drug transporter that the chloroquine uptake by *P. falciparum *infected erythrocyte is temperature-dependent and saturable and the existence of a specific parasite-encoded protein that facilitates chloroquine uptake. Although, there was no report in the effect of temperature toward mefloquine, quinine and artesunate, high temperature might have some effects to drug transport or drug mechanism by direct or indirect way as shown in our study. Moreover, high temperature after new ring infection shows some effects only toward standard or laboratory strain K1 or 3D7 but not to field isolate.

In order to observe the effect of temperature and drug by mimicking the situation of treatment failure in malaria patients coped with fever and drug uptake, it was found that high temperature and chloroquine had no effect to the development of parasite with chloroquine resistant (K1 strain). However, high temperature and chloroquine had effect toward 3D7 and isolate PF91 development. After culture under high temperature with artesunate, the total parasite number of all strains was extremely decreased at phase B and the parasite was not found at phase C and D. No parasite was found at phase C in group with high temperature and artesunate. This might indicate that all parasites died at phase C.

High temperature coped with chloroquine promotes the expression of heat shock protein 70. The proteome extracted from K1 grown under heat shock with chloroquine, anti-*pf*Hsp70 interacted with four bands whereas anti-*pf*Hsp70 interacted with only one band in another strain under different condition. After protein analysis by MALDI-TOF peptide mass fingerprint, the 120 kDa band from all gels is identified as heat shock protein 70 or pfHSP70s. Higher molecular weight may be caused by the complex formation of heat shock protein as molecular chaperone. The 83 kDa, 60 kDa and 40 kDa hybridized band was identified as elongation factor 1α, *pf*Hsp 86 and phosphoethanolamine *N*-methyltransferase, respectively.

## Competing interests

The authors declare that they have no competing interests.

## Authors' contributions

RA conceived of the study, participated in its design, interpretation of the results and drafted the manuscript. SS co-conceived of the study, participated in the study design, interpretation of the results and carried out all experiments. KN participated in the statistical analysis and its co-interpretation. RU participated in the design of the study, interpretation of the data and participated in the final revision. MM participated in the study design and interpretation of the results. PA co-performed the western blot analysis. WC partcipated in the drug sensitivity testing. All authors have read and approved the final manuscript.

## References

[B1] Hyde JE (2002). Mechanisms of resistance of *Plasmodium falciparum *to antimalarial drugs. Microbes Infect.

[B2] Anderson RM, May RM, Gupta S (1989). Non-linear phenomena in host-parasite interactions. Parasitology.

[B3] Long HY, Lell B, Dietz K, Kremsner PG (2001). *Plasmodium falciparum*: *in vitro *growth inhibition by febrile temperature. Parasitol Res.

[B4] Kwiatkowski D (1989). Febrile temperatures can synchronize the growth of *Plasmodium falciparum in vitro*. J Exp Med.

[B5] Pavithra SR, Banumathy G, Joy O, Singh V, Tatu U (2004). Recurrent fever promotes *Plasmodim falciparum *development in human erythrocytes. J Biol Chem.

[B6] Sharma Y (1992). Structure and possible function of heat-shock protein in *falciparum *malaria. Comp Biochem Physiol B.

[B7] Biswas S, Sharama Y (1994). Enhanced expression of *P. falciparum *heat shock protein *Pf*hsp 70-1 at higher temperatures and parasites survival. FEMS Microbiol Lett.

[B8] Joshi B, Biswas S, Sharama Y (1992). Effects of heat-shock on *P. falciparum *viability growth and expression of the heat-shock *Pf*HSP 70-1 gene. FEB Lett.

[B9] Trager W, Jensen JB (1976). malaria parasites in continuous culture. Science.

[B10] Jensen JB, Trager W (1977). *Plasmodium falciparum *in culture: use of outdated erthrocytes and description of the candle jar method. J Parasitol.

[B11] Desjardins RE, Canfield CJ, Haynes JD, Chulay JD (1979). Quantitative assessment of antimalarial activity *in vitro *by a semiautomated microdilution technique. Antimicrob Agents Chemother.

[B12] Pickard AL, Wongsrichanalai C, Purfield A, Kamwendo D, Emery K, Zalewski C, Kawamoto F, Miller RS, Meshnick SR (2003). Resistantance to antimarials in Southeast Asia and genetic polymorpisms in *pfmdr1*. Antimicrob Agent Chemoth.

[B13] Harinasuta T, Migasena S, Bunnag D (1962). Chloroquine resistantance in Thailand. Proceedings of the UNESCO First Regional Symposium on Scientific Knowledge of Tropical Parasites: Singapore.

[B14] Udomsangpetch R, Pipitaporn B, Silamut K, Pinches R, Kyes S, Looareesuwan S, Newbold C, White NJ (2002). Febrile temperatures induce cytoadherence of ring-stage *Plasmodium falciparum*-infected erythrocytes. Proc Natl Acad Sci USA.

[B15] Oakley MS, Kumar S, Anantharaman V, Zheng H, Mahajan B, Haynes JD, Moch JK, Fairhurst R, McCutchan TF, Aravind L (2007). Molecular factors and biochemical pathways induced by febrile temperature in intraerythrocytic *Plasmodium falciparum *parasites. Infect Immun.

[B16] Sanchez  CP, Wunsch S, Lanzer M (1997). Identification of chloroquine importer in *Plasmodium falciparum*. J Biol Chem.

[B17] Sargent TJ, Marti M, Caler E, Carlton JM, Simpson K, Speed TP, Cowman AF (2006). Lineage-specific expansion of proteins exported to erythrocytes in malaria parasites. Genome Biol.

[B18] Llya S, David G (2004). Novel regulation factor of HSF-1 activation: facts and perspectives regarding their involvement in the age-associated attenuation of heat shock response. Mech Ageing Dev.

[B19] Gowrishankar B, Varsha S, Pavithra SR, Tatu U (2003). Heat shock protein 90 function is essential for *Plasmodium falciparum *growth in human erythrocytes. J Biol Chem.

[B20] Morishiqe T, Tsujita T, Yamada Y, Sato F (2000). Molecular characterization of the S-adenosyl-L-methionine: 3'-hydroxy-N-methylcoclaurine 4'-O-methyltransferase involved in isoquinoline alkaloid biosynthesis in *Coptis japonica*. J Biol Chem.

[B21] Stadler R, Zenk MH (1993). The purification and characterization of a unique cytochrome P-450 enzyme from *Berberis stolonifer *a plant cell cultures. J Biol Chem.

